# Pharmacological Treatments and Therapeutic Drug Monitoring in Patients with Chronic Pain

**DOI:** 10.3390/pharmaceutics15082088

**Published:** 2023-08-05

**Authors:** Federica De Rosa, Bruno Giannatiempo, Bruno Charlier, Albino Coglianese, Francesca Mensitieri, Giulia Gaudino, Armando Cozzolino, Amelia Filippelli, Ornella Piazza, Fabrizio Dal Piaz, Viviana Izzo

**Affiliations:** 1 Department of Medicine, Surgery and Dentistry, Postgraduate School of Clinical Pharmacology and Toxicology, University of Salerno, 84084 Fisciano, Italy; fderosa@unisa.it (F.D.R.); b.giannatiempo1@studenti.unisa.it (B.G.); bcharlier@unisa.it (B.C.); dr.armando.cozzolino@gmail.com (A.C.); afilippelli@unisa.it (A.F.); 2 University Hospital “San Giovanni di Dio e Ruggi d’Aragona”, 84131 Salerno, Italy; albino.cog@gmail.com (A.C.); opiazza@unisa.it (O.P.); 3 Department of Medicine, Surgery and Dentistry, Postgraduate School of Clinical Pathology and Clinical Biochemistry, University of Salerno, 84084 Fisciano, Italy; 4 Department of Medicine, Surgery and Dentistry “Scuola Medica Salernitana”, University of Salerno, 84084 Fisciano, Italy; fmensitieri@unisa.it (F.M.); ggaudino@unisa.it (G.G.)

**Keywords:** therapeutic drug monitoring, opioids, chronic pain, pain management, analgesic drugs, morphine, relief, pain control, analgesia

## Abstract

Pain is an unpleasant sensory and emotional experience that affects every aspect of a patient’s life and which may be treated through different pharmacological and non-pharmacological approaches. Analgesics are the drugs most commonly used to treat pain, and in specific situations, the use of opioids may be considered with caution. These drugs, in fact, do not always induce optimal analgesia in patients, and several problems are associated with their use. The purpose of this narrative review is to describe the pharmacological approaches currently used for the management of chronic pain. We review several aspects, from the pain-scale-based methods currently available to assess the type and intensity of pain, to the most frequently administered drugs (non-narcotic analgesics and narcotic analgesics), whose pharmacological characteristics are briefly reported. Overall, we attempt to provide an overview of different pharmacological treatments while also illustrating the relevant guidelines and indications. We then report the strategies that may be used to reduce problems related to opioid use. Specifically, we focus our attention on therapeutic drug monitoring (TDM), a tool that could help clinicians select the most suitable drug and dose to be used for each patient. The actual potential of using TDM to optimize and personalize opioid-based pain treatments is finally discussed based on recent scientific reports.

## 1. Pain Definition, Classification and Action Mechanism

In 1978, the International Association for the Study of Pain (IASP) defined pain as “an unpleasant sensory and emotional experience associated with actual or potential tissue damage or described in terms of such damage” [1,2]. This definition is currently used by several professional, governmental, and non-governmental organizations, including the World Health Organization (WHO) [3,4,5,6,7,8,9,10,11,12,13]. The recent IASP definition also involved populations such as infants and the elderly and emphasized verbal self-report, including of cognitive and social factors, as an integral part of the pain experience [14,15,16].

**Figure 1:**
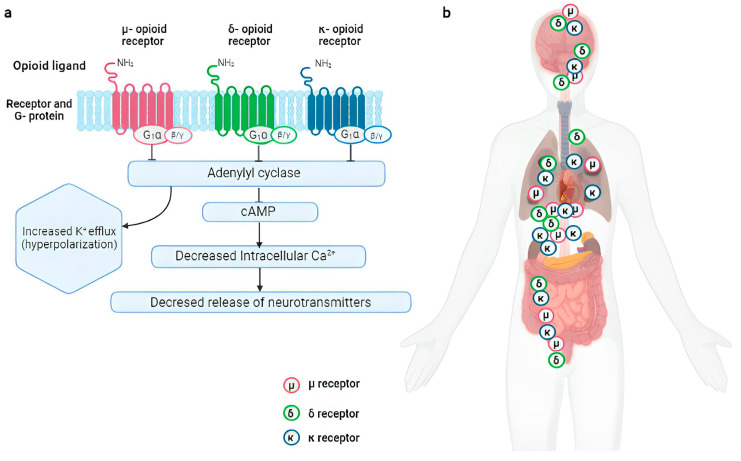
Opioid receptors: mechanism and distribution. (**a**) Schematic representation of the mechanism of action of opioid receptors; (**b**) distribution of opioid receptors (Created with BioRender.com).

Pain sensation is regulated by opioid receptors (ORs), which are G protein-coupled receptors (GPCR) of the G0/Gi inhibitory type. After the opioid agonist binds to the N-terminal extracellular domain of these receptors, adenylate cyclase activity is inhibited, and the formation of cAMP is reduced [17,18,19,20,21]. Opioids (either endogenous or exogenous) carry out various functional responses through the interaction between different classes of receptors, which may be co-stimulated [18,19]. The five currently known types of ORs are µ receptors (also called MORs), κ receptors (also called KORs), δ receptors (also called DORs), nociceptive receptors (also called NORs), and ζ receptors (also called ZORs) [17]. Of these receptors, only µ, δ, and κ are involved in pain pathways; these receptors exist as several subtypes, including µ1, µ2, µ3, κ1, κ2, κ3, δ1, and δ2 [17,18] (Figure 1).

In more detail, MORs are bound to the endogenous ligands beta-endorphin and endomorphin 1 and 2. The µ2 receptor is involved in the mechanisms of euphoria, addiction, respiratory depression, miosis, and decreased motility/constipation of the digestive tract, whereas the µ3 receptor is responsible for vasodilation. Dynorphins A and B bind to KORs, inducing analgesia, diuresis, and dysphoria. Enkephalins bind to DORs; indeed, they play a role in analgesia and in the reduction of gastric motility. The NORs cause analgesia and hyperalgesia depending on their location. The ζ receptors mediate the activity of the Met5-enkephalin, a regulating peptide acting as an opioid growth factor in developing cells.

The central nervous system (CNS) includes a high concentration of ORs in the periaqueductal gray, locus coeruleus (LC), ventral rostral medulla, and the gelatinous substance of the dorsal horn of the spinal cord. Peripheral receptors detect pain stimuli, and impulses are carried to the dorsal horn of the spinal cord for transmission to higher centers in the brain [19]. Activation of MORs by an opioid agonist in the midbrain causes the formation of descending inhibitory impulses mediated through inhibition of γ-aminobutyric acid (GABA) interneurons to the periaqueductal gray, which stimulates descending inhibitory neurons that activate enkephalin-containing neurons (the latter are connected directly to the dorsal horn), decreasing the nociceptive transmission from the periphery to the thalamus. The administration of exogenous opioids causes analgesia by acting on the substantia gelatinosa of the dorsal horn and on the peripheral afferent nerves [19]. The activation of MORs reduces the central response to stress through the inhibition of norepinephrine (NE) secretion from the LC, which is regulated by corticotropin-releasing hormone (CRH). The mechanism underlying the CRH activation of LC neurons is unknown; CRH receptors are GPCR and activate adenylate cyclase, but coupling with other second messenger cascades has also been described [22]. Thus, MORs are critical for stress recovery, which is confirmed by the reduction in the risk of developing post-traumatic stress disorder (PTSD) shown in studies describing the beneficial effect of morphine administration after a stressful event [19]. Furthermore, MORs, through the inhibition of GABA secretion, induce the release of dopamine, which is responsible for the rewarding effects produced by opioids administration [19,23]. Opioid receptors are present in the respiratory centers located in the cerebral cortex, in the thalamus, in the chemoreceptors of the carotid and vagus bodies, and in the mechanoreceptors of airways and lungs. Their stimulation can lead to hypercapnia and hypoxia [19,24,25]. ORs are present in the autonomic nervous system, especially in the gastrointestinal tract, where their activation causes a slowdown of peristalsis mediated by the inhibition of acetylcholine (ACh) release on myenteric neurons and via partial inhibition of purines and nitric oxide release from inhibitory motor neurons [26]. Their stimulation decreases the secretion of chloride, with consequent passive movement of water towards the intestinal lumen leading to the formation of hard stools and constipation [20,26,27]. The activation of the ORs present in heart tissue leads to hyperpolarization of the membranes and the activation of the vagus nerve, resulting in peripheral vasodilation and bradycardia, which ultimately cause hypotension [28]. Stimulation of ORs located in the hypothalamus inhibits the release of Gonadotropin Releasing Hormone (GnRH), reducing the secretion of luteinizing hormone (LH) and Follicle-Stimulating Hormone (FSH). Hence, chronic activation of these receptors leads to osteoporosis and sexual dysfunction, including decreased libido, infertility, and increased bone fragility. These receptors in the hypothalamus cause a decreased activity of the hypothalamic–pituitary–adrenal axis, resulting in low ACTH and cortisol levels. Low cortisol levels may clinically present with nonspecific symptoms such as anorexia, nausea, vomiting, abdominal pain, weakness, fatigue, lethargy, and fever [29]. Immune cells also have opioid receptors. Activation of the latter in natural killer cells (NK) and phagocytes inhibits their function, leading to a reduction in the immune response and a slowing of the healing process [19,30]. Lastly, activation of ORs in the reticular formation increases the duration of light sleep and decreases that of deep and REM sleep [29].

The evaluation of clinical pain is based on several features, including location and irradiation, aggravating or relief factors, and interactions with other psychological symptoms. Furthermore, the pain’s intensity (mild, moderate, or severe) and persistence (acute, chronic or breakthrough pain) is considered [31]. Based on these features, a diagnostic hypothesis can be generated along with the identification of one of the following four etiological mechanisms:

- Nociceptive: pain generated by tissue damage following an injurious event [32].
- Neuropathic: pain caused by damage or dysfunction of the peripheral or central nervous system [33].
- Nociplastic: pain induced by activation of nociception in the absence of damage tissue, real or potential [34].
- Mixed: pain presenting a complex overlap of components previously described in any combination [35].

In clinical practice, pain is evaluated using different scales, which quantify ache and are used to evaluate treatment efficacy. Pain intensity is the more frequently assessed aspect in clinical and research settings [36,37]. In most instances, four types of rating scales are used, each with its strengths and weaknesses: Numerical Rating Scales (NRS), Visual Analogue Scales (VAS), Verbal Rating Scales (VRS) and Facies Pain Scale (FPS) [38].

In 1986, for the first time, the WHO proposed the first edition of “Cancer Pain Relief”, which presented a methodological strategy mainly based on a drug program specifically intended to treat cancer pain [39]. Since then, different books and guidelines have been released and different studies have focused on approaches to treat different kinds of pain [40,41,42,43,44,45,46,47,48,49,50,51,52]. Indeed, since cancer is often incurable, pain management becomes the primary goal of treatment [40,47].

**Figure 2:**
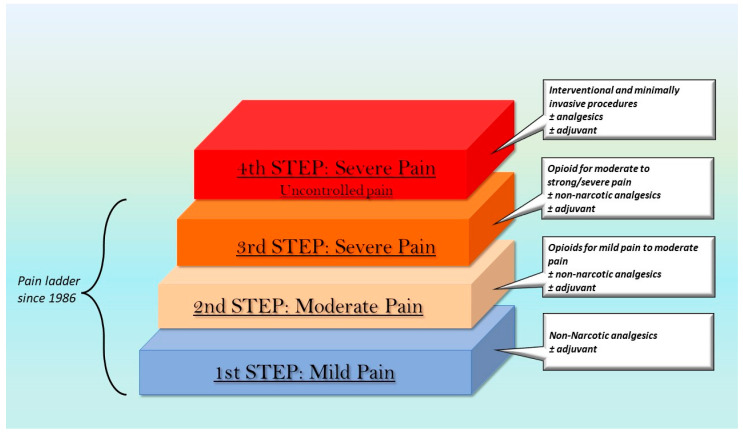
Evolution of pain ladders: steps 1, 2 and 3 are in the first edition of *Cancer Pain Relief*, published by WHO [39]; step 4 has been introduced for situations in which available pharmacological approaches used to control pain fail to be effective [53,56,59].

The WHO document specifically concerned cancer pain; gradually, however, further research has paid attention to the treatment of different types of chronic pain [48,49,50,51,52]. Originally, the pain ladder consisted of three steps, each associated with a pain intensity level and related drug treatment options. In the first step, there were mild pain suffering patients, for whom analgesic non-narcotic drugs were suggested, while the last step was dedicated to severe pain, which may be treated with strong opioids [39,40,53,54]. Over the years, the scale has undergone several changes and it is currently applied not only to cancer pain but also to the treatment of several forms of chronic pain. Different authors recommend adding a fourth step (Figure 2) concerning persistent pain and related treatment with interventional and minimally invasive procedures (e.g., epidural analgesia, intrathecal administration with and without pumps, nerve blocks, and ablative procedures) [53,55,56,57,58].

Since it is not always possible to recognize and treat the primary cause of chronic pain rapidly, pain relief may represent the main target of therapeutic treatment in the clinical evaluation process. The central point of chronic pain therapy is often represented by the pharmacological treatment based on the use of analgesic drugs, which can be associated with a surgical, neurosurgical, psychological or radiotherapeutic approach. The whole range of drugs used varies widely, as does their availability in different countries, and their use must also be considered with regard to the concurrent pathological conditions [39,40].

## 2. The Pharmacological Approach

Since the first publication of “Cancer pain relief”, it has become quite evident that pain management, regardless of the type of pain, should be tailored to the individual patient [40,60]. Moreover, the progress of knowledge on pain mechanisms, the results derived from clinical trials, and the development of new medical technologies have allowed researchers to improve the efficacy of the available therapies [60,61,62,63,64].

The drugs used for various types of pain are grouped in different classes.

### 2.1. Adjuvant Drugs

Cancer pain is multifactorial and often involves nociceptive and neuropathic subtypes of pain, as previously discussed. WHO suggests combining analgesics (non-opioid and opioid) with adjuvant analgesics, also called co-analgesics [39,40]. These drugs do not have analgesia as their primary indication but can be useful in the management of chronic pain [65]. The combination of adjuvants and opioids has been widely used to treat cancer pain syndromes, but it is also used for other types of pain [65]. The most appropriate drug combination is selected based on the type of pain and its action mechanism. The adjuvant drugs initially indicated by the WHO are: antidepressants, anticonvulsants, local anesthetics, and steroids, to which bisphosphonates, calcitonin and cannabinoids have also been added [39,40,65]. Adjuvant analgesics should be initially administered in low doses, which may increase if efficient therapeutic effect is not obtained [65].

Antidepressants (particularly tricyclic antidepressants and serotonin and norepinephrine reuptake inhibitors) have traditionally been used to treat neuropathic pain associated with diabetic neuropathy and post herpetic neuralgia [39,40,65]. These drugs also treat medical conditions, such as depression, anxiety states and insomnia, which are important factors in pain management [40,65].

Anticonvulsant drugs treat neuropathic symptoms that may also be secondary to nerve injury [65]. In fact, by modulating voltage-gated ion channels (sodium and/or calcium channels), these drugs modify neuronal activity and decrease pain intensity [39,40,65].

Bisphosphonates are recommended for bone pain in case of bone metastases and multiple myeloma. Local anesthetics may be useful in the management of cancer pain, especially in hospitalized patients [65]. They have antinociceptive properties and inhibit the proliferation of mesenchymal stem cells [66,67].

Corticosteroids are used for the treatment of bone pain associated with metastases, malignant epidural compression, and cerebral edema secondary to brain injury, but also to treat neuropathic pain in which peripheral nerves have been compressed by tumors [39,40,65].

Regarding cannabinoids, they seem to improve some pathological aspects of patients suffering from chronic pain (such as sleep and quality of life) [67]. Different studies have investigated the analgesic effects of the marijuana plant (cannabis) or cannabinoids in different types of pain [68,69,70]. It is important to underline, however, that as of 2021, after two and a half years’ review work, the International Association for the Study of Pain (IASP), has discouraged the use of cannabinoids for pain treatment [70]. Although there are several preclinical data supporting the hypothesis of cannabinoid analgesia, robust clinical evidence is still lacking [70].

### 2.2. Non-Narcotic Analgesics

Non-narcotic analgesics are drugs of first choice in the treatment of mild to moderate pain. They do not generally exert undesired effects such as physical addiction or tolerance.

#### 2.2.1. Nonsteroidal Anti-Inflammatory Drugs (NSAIDs)

Non-steroidal anti-inflammatory drugs (NSAIDs) block cyclooxygenase (COX) by inhibiting the biosynthesis of prostaglandins (PGs), especially prostaglandin E2 (PGE2) and prostaglandin I2 (PGI2) and thromboxane [39,40,71]. Their use is encouraged when pain depends on a strong action exerted by prostaglandins and bone metastases, since COX2 is expressed in tumor cells and in the surrounding macrophages [72]. NSAIDs may be useful to treat acute post-operative pain [73], chronic pain due to arthrosis [74] and chronic non-cancerous pain in children and adolescents [75].

NSAIDs may be used alone or in addition to opioids in moderate and severe cancer pain. Clinicians, in an effort to improve analgesic control, often add a non-opioid to an opioid to combine drugs with a different mechanism of action, thus effectively reducing opioid requirements and minimizing their adverse effects [76]. The main NSAIDs used for pain management are acetylsalicylic acid (ASA), ibuprofen, naproxen, and celecoxib (Table 1). Elderly patients or patients with kidney, liver, or heart disease should use NSAIDs with caution because side effects like cardiotoxicity, nephrotoxicity, hepatotoxicity, and impaired platelet function may occur [71].

#### 2.2.2. Paracetamol

Paracetamol is a para-aminophenol derivative [71]. The exact action mechanism of paracetamol is still unclear; Graham et al. suggest that, like NSAIDs, it interferes with prostaglandin (PG) production by weakly and indirectly inhibiting COXs and blocking the formation of phenoxy radicals essential for the cyclooxygenase activity, thus hampering PG biosynthesis [77]. Paracetamol seems to display COX2 selectivity, given its poor antiplatelet activity and good gastrointestinal tolerance [71], and it is characterized by a safety profile which, although it does not have a precise anti-inflammatory action, has analgesic and antipyretic properties [71,77].

Paracetamol is the only non-opioid analgesic recommended for pregnant women for limited use over time [78,79]. It has a good safety and tolerability profile within the recommended dose of up to 4 g per day. Paracetamol overdose induces hepatic injury and renal toxicity [80]. Limited use is recommended for the treatment of acute and chronic non-cancer mild and moderate pain, except for back pain and some types of osteoarthritis [81] (Table 1). The use of paracetamol to treat lower back pain is controversial. It has been recommended over both non-selective NSAIDs and selective COX-2 inhibitors as the preferred initial analgesic in osteoarthritis and lower back pain because of its superior tolerance and in terms of cost–benefit [77]. However, the Cochrane review has not reported conclusive data to support the effectiveness of paracetamol for acute lower back pain in the immediate and short term [81].

**Table 1:** The list of major non-narcotic analgesic drugs including paracetamol and non-steroidal anti-inflammatory drugs (NSAIDs).

Basic Drug	Use	Mechanism of Action
Acetylsalicylic acid (ASA)	It relieves mild to moderate acute pain [82].	Non selective non-steroidal anti-inflammatory drugs (nsNSAIDs) [71].
Ibuprofen	It is preferable as a drug of first choice to provide relief from musculoskeletal pain in children. It is used in several clinical conditions, such as dysmenorrhea, dental pain, headache and migraine, soft tissue pain, and fever [83].	Non selective non-steroidal anti-inflammatory drugs (nsNSAIDs) [71].
Naproxen	It is used for post operative pain/acute pain [84]. It is the first-line treatment for acute gouty arthritis, osteoarthritis, musculoskeletal pain, inflammation, and dysmenorrhea [85].	Non selective non-steroidal anti-inflammatory drugs (nsNSAIDs) [71].
Celecoxib	It is administered before surgery because it decreases the post operative pain intensity of arthroscopy [86]. It seems to have a superior efficacy compared with paracetamol in chronic nonspecific lower back pain [87].	Selective cyclo-oxygenase 2 NSAIDs (COXIBs) [71].
Paracetamol	It provides pain relief in chronic osteoarthritic pain and lower back pain [77]. Furthermore, it may be used in combination with opioids for cancer pain [77]. It is the first-line treatment for the majority of mild to moderate acute pains [88]. Paracetamol is also effective for acute renal colic pain [81,89,90,91].	Partial Selective cyclo-oxygenase 2 NSAIDs (COXIBs) [71].

### 2.3. Opioids

Opioids are the oldest drugs used for pain treatment and can be classified according to their use for mild to moderate pain or for moderate to severe pain [39,40]. Opioids are available in different pharmaceutical formulations; oral administrations are the most recommended for the management of mild to moderate pain, while parenteral formulations are recommended for the management and relief of severe pain, when pain is not controlled, due to their rapid onset [39,40,92].

Endogenous peptides (endorphins, enkephalins, dynorphins and nociceptins) are produced in response to painful stimuli. These compounds bind to their specific receptors located in the CNS and in peripheral tissues [92,93]. The analgesic activity of opioids is due to the presence of the receptors on the pain modulation pathways. Furthermore, studies from the 1990s identified the presence of opioid receptors on nociceptive C fibers and A δ fibers [94]. At this level, the interaction between opioids and ORs results in a blockade of pain neurotransmitters, releasing glutamate, substance p, calcitonin gene-related peptide, etc. [95,96]. Another pain mechanism action is related to the inhibition of GABA release, which stimulates dopamine delivery involved in the modulation of descending pain [93,97]. Consequently, an inhibition of the excitatory currents is obtained, resulting in reduced transmission of nociceptive stimuli to all levels of the CNS and a deep reduced pain perception [93,95,96]. Commonly available opioid drugs (morphine, codeine, methadone, fentanyl, and their derivatives) are mainly agonists of a particular receptor, the µ receptor [96]. There are also partial agonist opioids, like buprenorphine, and agonist–antagonist opioids like pentazocine and butorphanol [93].

To select the most suitable opioid for pain relief, aspects including patient age, the type and nature of the pain, pharmacokinetic considerations (route of administration, absorption, distribution, desired onset or duration, sustained-release formulations, metabolism, excretion) and side effects depending on dosage and redistribution [96,97,98,99] should be considered. Moreover, some opioids have high oral bioavailability, while others, such as buprenorphine and fentanyl, have poor and highly variable bioavailability [99]. Parenteral formulations may be preferred when rapid pain relief is required [98,99].

Even though opioids have numerous advantages, they also induce the development of tolerance, meaning that persistent use may cause a reduction in the efficacy and the duration of action, which may in turn require an adjustment of dosage to achieve the same analgesic effect. In addition, abrupt cessation of chronic opioid use during continued treatment produces an aversive withdrawal syndrome. It consists of signs and symptoms including stomach cramps, diarrhea, rhinorrhea, sweating, elevated heart rate, increased blood pressure, irritability, dysphoria, hyperalgesia, and insomnia. [100,101].

#### 2.3.1. Opioids for Mild to Moderate Pain

Weak opioids (codeine and tramadol) are analgesics indicated in step two of the WHO analgesic ladder (opioids for mild to moderate pain), which are prescribed when non-opioids fail to provide adequate relief in patients with moderate pain [39,40,102].

**Figure 3:**
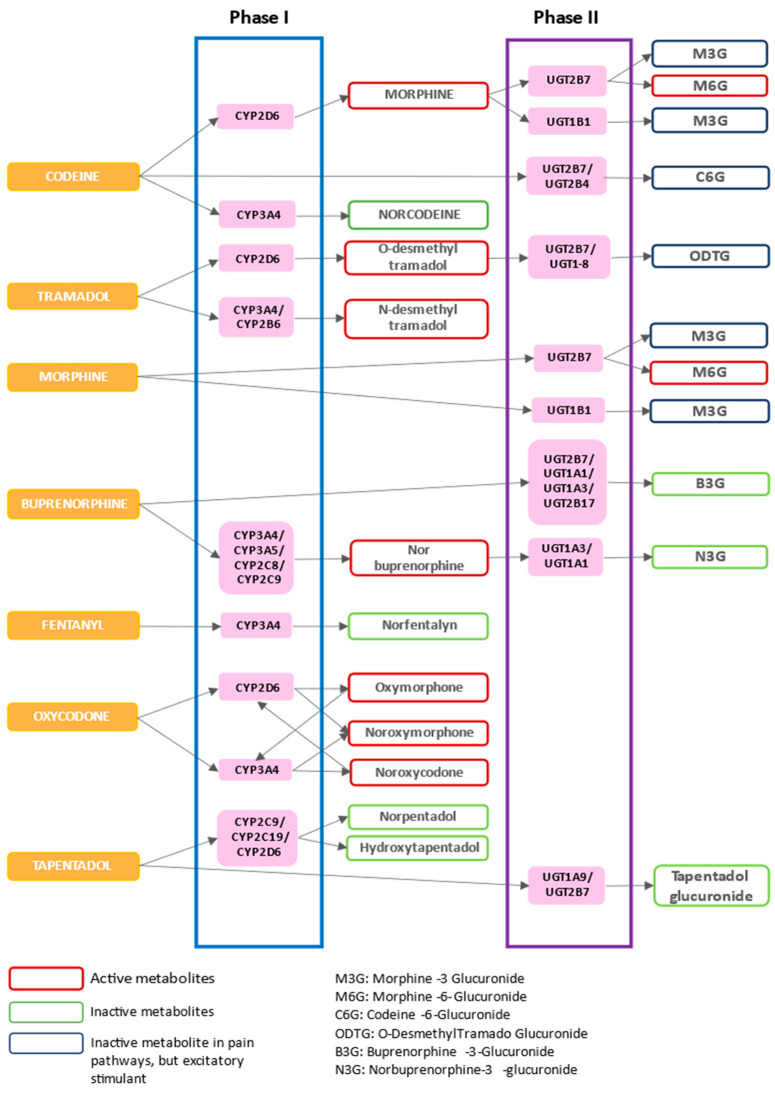
Metabolic pathways of opioid analgesics in the liver (CYP: cytochrome P450; UGT: uridine diphosphate glucuronosyltransferase).

Codeine is used to treat mild to moderate chronic pain [103], showing a weak affinity to µ receptors compared to morphine. It has an apparent volume of distribution of 3 to 6 L/kg and its main metabolites—morphine and codeine-6-glucuronide (C6G)—are the major effectors of the analgesic activity [93,103]. Cytochrome P450 isoenzyme 2D6 (CYP2D6) is the enzyme responsible for the transformation of codeine to morphine (about 5–15%), and the CYP3A4 converts codeine to norcodeine (about 10–15%), while uridine diphosphate glucuronyltransferase (UGT) 2B7 is the phase II metabolism enzyme responsible for producing C6G (about 50–70%), morphine-3-glucuronide (M3G) and morphine-6-glucuronide (M6G) from morphine (Figure 3) [103,104]. It has a half-life of 3 h and 90% of the codeine is excreted through the kidney [104]. Constipation is one of the most common adverse effects of this drug [104]. Codeine is often used in combination with paracetamol to take advantage of the synergistic action of the two different active compounds, and is used to treat pain of different origins (Table 2), especially in elderly patients and in those undergoing long-term treatment [105].

Tramadol is a synthetic opioid derived from morphine, which has multiple binding targets. It binds not only to opioid receptors, but also to different monoaminergic, serotonergic, and ion channel (muscarinic, nicotinic and K^+^ channels) receptors [106]. Tramadol exists as a racemic mixture of two pharmacologically active enantiomers: (+)-tramadol inhibits the reuptake of serotonin and (−)-tramadol inhibits the reuptake of norepinephrine [107]; in addition (+)-tramadol and its primary metabolite, (+)-*O*-desmethyltramadol (M1), are µ receptor agonists [107]. Moreover, tramadol binds weakly to κ-, δ- and μ-opioid receptors, inhibiting the reuptake of NE and 5-Hydroxytryptamine (5-HT) and the descending pathways of pain. It also blocks sodium and heat-sensitive transient receptor potential V1 (TRPV1) channels, as observed in vitro [106]. In addition, several studies demonstrated the anti-inflammatory effects of tramadol, which reduces the levels of PGE2 and tumor necrosis factor (TNF-α) in the cerebrospinal fluid [108]. This drug undergoes partial first pass metabolism in the liver by *N*- and *O*-demethylation and conjugation. *O*-demethylation of tramadol produces *O*-desmethyltramadol (M1) and is catalyzed by CYP2D6, whereas *N*-demethylation to *N*-desmethyltramadol (M2) is catalyzed by CYP3A4 and CYP2B6 [107,108,109,110] (Figure 3). Tramadol metabolites are mainly (about 90%) excreted through the kidneys and the remaining 10% undergo fecal excretion [107,108,109,110]. Tramadol has a half-life of 5–6 h, while metabolite M1 has a half-life of 8 h [109]. This opioid produces satisfactory analgesia against various types of pain, alone or in combination with non-opioid analgesics. It may be used, when other pharmacological and/or non-pharmacological options fail to succeed, for the treatment of lower back pain, neuropathic pain, and pain related to osteoarthritis and rheumatoid arthritis [111,112]. In addition, some evidence of its efficacy can be retrieved from the treatment of acute and postoperative pain [113,114,115] (Table 2).

#### 2.3.2. Opioids for Moderate to Strong/Severe Pain

Recently, several active opioid compounds have become available with different formulations, dosages, and routes of administration [99]. Strong opioids, included in the third level of the WHO ladder, may be used to treat severe cancer pain [38,39]. In this review, we have focused our attention on the most commonly used drugs, including morphine, buprenorphine, fentanyl, oxycodone and tapentadol (Table 2).

Morphine is one of the oldest known drugs [116]. It shows a strong binding to the µ receptor, while its binding to the δ and κ receptors is weaker [117]. It is mainly metabolized by UGT2B7 in M3G and M6G [103,104,118] (Figure 3). Morphine-3-glucuronide is inactive as an analgesic but has excitatory effects on the CSN [118,119], while M6G is the analgesic metabolite [114,120]. Morphine can be administered through different routes: intravenous, intramuscular, subcutaneous, oral, rectal, epidural, and intrathecal [121]. Intrathecal morphine is recommended for cancer pain and for non-cancer severe uncontrolled neuropathic and nociceptive pain [122,123]. This direct administration allows us to lower the drug dose, thus reducing its adverse effects [122]. Epidural morphine is used as an analgesic, in combination with other anesthetics, in several surgical procedures [124]. In 2004, the FDA approved the use of liposome-based epidural extended-release morphine (EREM), which triggers a 3 h-delayed concentration peak in the cerebrospinal fluid, thus providing pain relief even after 48 h from a single administration [125]. Rectal administration of morphine (through suppositories or solutions) is used for pain relief, especially in children [126], but it is not suitable for treating acute pain. Indeed, rectal administration causes variable absorption rates and metabolite plasma levels [127]. Therefore, since 1986, the WHO has recommended the oral route of administration because it is effective, inexpensive and easy to apply, thanks to high patient compliance [39]. Oral morphine generally produces good pain relief for patients suffering from moderate to severe pain [39,40,42]. It is currently available in several formulations that release morphine over various periods of time [42]. Immediate-release morphine is rapidly absorbed and should usually be taken every four hours. In alternative, modified-release tablets release morphine more slowly, so that they may be administered only twice or once a day [42]. Subcutaneous, intravenous, and intramuscular administrations are an effective alternative to the oral route in case of confusion, altered mental status, and persistent pain exacerbations in cancer pain patients [128]. Furthermore, these routes of administration are indicated in patients who need a rapid dose increase [129] to treat different types of pain [130,131,132]. The intravenous route is also exploited, particularly for postoperative analgesia [127,133]. Finally, recent studies have shown that morphine not only plays a role in cancer relief but may also be involved in the regulation of tumor growth, angiogenesis, metastasis, inflammation and immunity. It is worth noting, however, that these studies are highly controversial, mainly because the action mechanisms of the drug are still in part unclear and undefined [134]. Therefore, more significant and extensive evidence is required to redefine morphine function in tumor processes [134].

Buprenorphine has unique pharmacodynamic properties among all opioids because it can behave as an agonist, partial agonist, and antagonist of different ORs. It is a weak κ receptor antagonist and δ receptor agonist [135]. Buprenorphine is a partial agonist of the µ receptor; indeed, even if it has a high affinity for this receptor, the maximum effect produced is lower than that of pure agonists [136]. This high affinity makes it difficult to displace the drug from the µ receptor, thus blocking other opioids [136,137]. Buprenorphine is extensively metabolized to norbuprenorphine by CYP3A4/CYP3A5, with contributions from CYP2C8 and CYP2C9. Norbuprenorphine undergoes glucuronidation by UGT1A3 and UGT1A1 to norbuprenorphine-3-glucuronide (N3G), while buprenorphine can be inactivated by UGT2B7, UGT1A1, UGT1A3 and 2B17 to buprenorphine-3-glucuronide [136,138,139] (Figure 3). Given its slow kinetics of dissociation from the receptor, this drug exerts a prolonged action, which allows for a single daily administration [136]. Considering these multiple neurophysiological properties, buprenorphine may be used in opioid addiction; indeed, a strong agonist (such as heroin) can be displaced by buprenorphine thanks to its high affinity [135,136,137,138]. The oral bioavailability of buprenorphine is very low and highly variable [100]; buccal film and sublingual tablets are used to treat breakthrough pain (BTP) [140], while a transdermal patch is used for chronic pain relief [135]. Conversely, in perioperative settings, subdermal or subcutaneous implants and intravenous (IV) or intramuscular (IM) injections are frequently used [135,141]. A combination of buprenorphine and naloxone (buprenorphine/naloxone in ratio 4:1) in the form of sublingual tablets is used for the treatment of opioid addiction [142] and, in recent years, it has increasingly been prescribed off-label for the management of chronic pain [142].

Fentanyl has a low affinity for δ and κ opioid receptors, but it has a high affinity for the µ receptor, of which it is an agonist [137,143]. It is mainly metabolized by CYP3A4 in norfentanyl, an inactive compound [144] (Figure 3). It is similar to morphine in its elimination/clearance half-life, but the onset and duration of its analgesic action is shorter [143]. Fentanyl lipophilicity allows it to rapidly cross the blood–brain barrier but is also responsible for its accumulation in body fat, which contributes to the short activity duration of this drug [143,144]. This characteristic lipophilicity affects the route of administration, the pharmacokinetics, and also the peculiar formulations that have been developed in recent years [143]. Indeed, fentanyl is available for intravenous, transdermal, and transmucosal administration [145]. The transdermal patch is also available to manage chronic non-cancer pain and chronic cancer pain [145]. The rapid onset transmucosal fentanyl preparations have been developed for BTP [145], while intravenous formulation is widely used for anesthesia and analgesia, often in operating rooms and intensive care units [143,145].

Oxycodone is an agonist of the μ opioid receptor, and it binds the δ and κ-opioid receptors [146,147]. Its binding affinity to the μ-opioid receptor is lower than that of morphine, but oxymorphone, the active metabolite of oxycodone, has a significantly greater affinity than the parent drug [146]. The liver enzyme CYP2D6 turns oxycodone to oxymorphone, while the CYP3A4 converts oxycodone in nor-oxycodone. The same CYP2D6 is responsible for converting noroxycodone to noroxymorphone [146] (Figure 2). Oxycodone has an oral bioavailability higher than 60% and is widely used in clinical practice to manage postoperative, neuropathic, and cancer pain [146,147,148,149]. Oxycodone is mainly used in the form of controlled-release tablets for chronic pain, whereas the immediate-release solution or tablets are used for acute pain or for BTP [146]. It is also available for intravenous, intramuscular, intranasal, subcutaneous, and rectal routes, which are good alternatives when opioids cannot be administered orally [146,148]. Moreover, oxycodone is often combined with non-narcotic analgesics (like paracetamol) with the purpose of increasing the synergistic effect, thus reducing opioid dosage and the consequent incidence of adverse events [150,151]. In several studies, the oxycodone–paracetamol oral combination has allowed an adequate analgesia management for moderate-severe cancer pain [150,151].

Tapentadol is a µ-opioid receptor agonist; it has lower binding affinity and is less potent than morphine, but it is also a strong NE reuptake inhibitor and a weak 5-HT reuptake inhibitor [152,153]. These mechanisms of action act synergically, providing a strong analgesic effect and overcoming opioid-related adverse events [152]. Unlike conventional opioids (e.g., oxycodone, morphine, tramadol), tapentadol has no active metabolites, it is primarily metabolized by UGT1A9 and UGT2B7 in tapentadol glucuronide [153]. In addition, phase I oxidative enzymes CYP2C9, CYP2C19 and CYP2D6 are involved in the production of nortapentadol and hydroxytapentadol [153] (Figure 3). Tapentadol has shown a favorable long-term safety profile in studies evaluating specific adverse events such as seizures, gastrointestinal events, hypertension, pulmonary dysfunction, serotonin syndrome, and endocrine toxicity [151]. Indeed, several clinical studies have confirmed the good tolerability profile of tapentadol and a lower risk for abuse [152,153,154,155,156]. Tapentadol is a drug that has recently been used in chronic therapies—for cancer and non-cancer pain—in different age groups, such as the elderly and children [152,155,156]. It is administered orally in immediate-release and extended-release formulations and is used for the treatment of chronic neuropathic and mixed pain [152,155,156]. It is often used in combination with anticonvulsant drugs (e.g., gabapentin or pregabalin) to treat severe and mixed neuropathic pain [155,156].

Information about the pharmacokinetic properties of opioid for moderate to severe pain is summarized in Table 3.

**Table 2:** Drug list of narcotic analgesics.

Basic Drug	Use	Mechanism of Action
Codeine	It is used for mild to moderate pain in the treatment of acute and chronic noncancer pain [103]. The combination paracetamol/codeine may be used to treat postoperative pain, osteoarthritis related pain, cancer pain and polytrauma pain [105].	Weak affinity to µ receptors [93,103].
Tramadol	It is used for mild to moderate pain alone or in combination with nonopioid analgesic drugs. Several studies show the efficacy for the treatment of lower back pain, neuropathic pain, pain related to osteoarthritis and rheumatoid arthritis, acute and postoperative pain [107,108,109,110,111,112,113,114,115].	Weak affinity to µ receptors, but it binds to monoaminergic, serotonergic receptors and ion channel receptors (muscarinic, nicotinic and K^+^ channels) [106]. It inhibits NE and 5-HT reuptake and it reduces the levels of PGE2 and TNF-α [106].
Morphine	It can be administered through different routes of administration: intravenous, intramuscular, subcutaneous, oral, rectal, epidural and intrathecal [121]. It is used for moderate to severe pain in the treatment of acute and chronic noncancer pain and cancer pain [39,40,41,42,122,123].	High affinity to the µ receptor, while the binding is weaker than the δ and κ receptors [117].
Buprenorphine	It is used in opioid addiction [135,136,137,138]. The oral forms are used to treat BTP [139]. The subdermal or subcutaneous implant, intravenous or intramuscular injections, and transdermal patches are used for the treatment of chronic noncancer pain and cancer pain [135]. Transdermal buprenorphine is not approved for children, while the parenteral form is frequently used in the perioperative setting [141].	It is a weak κ receptor antagonist and δ receptor agonist [135] and it is a partial (or low efficacy) agonist of the µ receptor [136].
Fentanyl	The patch is available for the management of chronic noncancer pain and chronic cancer pain [145]. Rapid onset transmucosal fentanyl preparations have been developed for BTP [145], while intravenous formulation is widely used for anesthesia and analgesia, often in operating rooms and intensive care units [143,145].	It has lower affinity for δ and κ opioid receptors, but it has high affinity for the µ receptor of which it is an agonist [137,143].
Oxycodone	It is widely used in clinical practice to control postoperative pain, neuropathic pain and cancer pain [148,149]. Oxycodone is mainly used in the form of controlled-release tablets for chronic pain, whereas the immediate-release solution and tablets are used for acute pain or for BTP [146]. It is also available for intravenous, intramuscular, intranasal, subcutaneous and rectal routes, which are good alternatives when opioids cannot be administered orally [146,148]. The oral combination oxycodone–paracetamol has shown an adequate analgesia management for moderate–severe cancer pain [150,151].	It is an agonist of μ opioid receptor and it also binds the δ and κ-opioid receptors [146,147].
Tapentadol	Unlike conventional opioids, it has shown a favorable long-term safety profile in studies evaluating specific adverse events such as seizures, gastrointestinal events, hypertension, pulmonary dysfunction, serotonin syndrome, and endocrine toxicity [152]. It is a drug that has recently been used in chronic therapies for both cancer and non-cancer pain, for different age groups, such as the elderly and children [152,155,156]. It is administered orally in immediate-release and extended-release formulations and is used for the treatment of chronic neuropathic and mixed pain [152,155,156]. It is often used in combination with anticonvulsant drugs (e.g., gabapentin or pregabalin) to treat severe and mixed neuropathic pain [155,156].	It is an agonist of µ-opioid receptor, but it is also a strong NE reuptake inhibitor and a weak 5-HT reuptake inhibitor [154,155].

**Table 3:** Pharmacokinetic properties of opioid for moderate to severe pain.

Drug	Volume of Distribution	Protein Binding	Clearance	Log P	Binding Affinity for Opioid Receptor (Ki) (Median)
Morphine	2.1–4.0 L/kg [157].	35%; 10% for M3G and 15% for M6G [120].	1600 mL/min (intravenous or subcutaneous) [158].	0.9 [159].	µ = 14 nM, ĸ = 47 nM, δ = 140 nM [117].
Oxycodone	2.6 L/kg [160].	45%, primarily serum albumin and, to a lesser extent, α_1_ acid glycoprotein. [161].	1400 mL/min [162].	0.7 [163].	μ = 18 ± 4 nM, δ = 958 ± 499 nM, κ = 677 ± 326 nM [146].
Buprenorphine	188–335 L [164].	96%, primarily to α- and β-globulin. [164].	1042–1280 mL/min [164].	4.5 [163].	μ = 0.2157 nM [165].
Fentanyl	4 L/kg [166].	80–85%. It is unclear whether fentanyl binds primarily to albumin (ALB) or α_1_ acid glycoprotein (AAG) [167].	500–1200 mL/min [168].	3.8 [163].	Μ = 1.35 nM [165].
Tapentadol	540 ± 98 L [169].	20% [169].	1530 ± 177 mL/min [170].	2.87 [171].	μ = 160 nM [172].

#### 2.3.3. Pharmacogenomics of Opioids

In pain therapy, failure to respond to drug treatment and the occurrence of adverse events have frequently been described. A possible reason for the great variability observed in opioid response may be related to the presence of genetic polymorphisms [173,174]. Polymorphisms of pharmacokinetic (which encode for phase I and phase II enzymes) and pharmacodynamic-related genes (such as OPRM1, which encodes the µ receptor) have been studied to identify potential explanation to the lack of pain control [173,174]. Indeed, the different genes encoding opioid receptors and those involved in opioid metabolism are characterized by a highly polymorphic nature [174].

As mentioned before, opioids are extensively metabolized in the liver, where the major phase I enzymes involved are CYP2D6, CYP3A4, and CYP3A5. Phase II enzymes include uridine UGTs [103,104,107,108,109,110,118,136,138,139,144,146,153]. The major metabolic pathways of previously described opioids are shown in Figure 3. Among the drugs considered, codeine, tramadol, buprenorphine, oxycodone, and morphine are those characterized by the presence of active metabolites. Hence, genetic polymorphisms affecting enzymes involved in the metabolism of these drugs can lead to altered plasmatic concentrations [175]. Reported variability of metabolites concentration in drug exposure translates into differences in drug efficacy and may affect the occurrence of adverse effects [175].

Approximately 10% of codeine is metabolized by CYP2D6 to morphine [103,104]. Tramadol is transformed into its active metabolite, *O*-desmethyltramadol (ODT) in the liver via the CYP2D6 enzyme [107,108,109,110]. CYP2D6 is highly polymorphic; in fact, more than 100 variants have been identified, which causes high variability in the enzymatic activity [176]. Indeed, the *Clinical Pharmacogenetics Implementation Consortium* (CPIC) guidelines suggest a careful analysis of this polymorphism when drugs such as codeine and tramadol are prescribed for long periods, as in the case of pain therapy [173].

Morphine is mainly metabolized in M3G and M6G by the UGT2B7 phase II enzyme in the liver, but UGT1A1 also plays a role in the glucuronidation of morphine to M3G. However, guidelines supporting pharmacogenetic testing to help guide morphine dose selection are currently lacking. This may be a consequence of the fact that no statistically significant correlations were found among the use of morphine, the occurrence of adverse effects, and the presence of genetic polymorphisms [173].

Oxycodone is mainly converted by CYP3A4/5 to noroxycodone, for more than 50% of the overall metabolic pathway, while the CYP2D6 is able to transform the drug into a more potent analgesic, oxymorphone [173,177]. Although several studies have demonstrated the influence of different polymorphisms on these enzymatic activities, no indication from the CPIC is available concerning the analysis of specific polymorphisms of oxycodone metabolizing enzymes [173].

Several case-control studies have investigated single-nucleotide polymorphisms (SNPs) in opioid receptors genes and their correlation with opioids addiction. However, these studies have often produced conflicting results. As an example, several studies showed that the presence of the G variant of OPRM1 (rs1799971) confers reduced analgesic effects on treatment with morphine, but the results of these trials were controversial [148,173,178]. Currently, guidelines from CPIC provide recommendations to tailor treatment only with codeine and tramadol metabolized by CYP2D6 (Table 4 and Table 5) [173,179]. However, extended guidelines for other opioids, which are more commonly used in real settings for pain management, are undoubtedly needed.

**Table 4:** Gene-specific information and correlations with metabolizer phenotypes and doses adjustment for codeine [179].

Phenotypes	Activity Score Range ^a^	Exemples of CYP2D6 Diplotypes	Implications	Recommendations
CYP2D6 ultrarapid metabolizer	>2.25	*1/*1 × N, *1/*2 × N, *2/*2 × N	Increased formation of morphine leading to higher risk of toxicity	Avoid codeine use because of potential for serious toxicity. If opioid use is warranted, consider a non-tramadol opioid.
CYP2D6 normal metabolizer	1.25 ≤ × ≤ 2.25	*1/*10 *1/*41, *1/*9 *10/*41 × 3 *1/*1, *1/*2 *2×2/*10	Expected morphine Formation.	Use codeine label recommended age-specific or weight-specific dosing.
CYP2D6 intermediate metabolizer	0 < × < 1.25	*4/*10 *4/*41, *10/*10 *10/*41 *41/*41, *1/*5	Reduced morphine Formation.	Use codeine label recommended age-specific or weight-specific dosing. If no response and opioid use is warranted, consider a non-tramadol opioid.
CYP2D6 poor metabolizer	0	*3/*4, *4/*4, *5/*5, *5/*6	Greatly reduced morphine formation leading to diminished analgesia	Avoid codeine use due to the possibility of diminished analgesia. If opioid use is warranted, consider a non-tramadol opioid.

^a^ is the sum of the values assigned to each allele, which typically ranges from 0 to 3 but may exceed 3 in rare cases. The asterisk (*) symbol is the standard wording to define genotypes.

**Table 5:** Gene-specific information and correlations with metabolizer phenotypes and doses adjustment for tramadol [179].

Phenotypes	Activity Score Range ^a^	Examples of CYP2D6 Diplotypes	Implications	Recommendations
CYP2D6 ultrarapid metabolizer	>2.25	*1/*1 × N, *1/*2 × N, *2/*2 × N	Increased formation of O-desmethyltramadol (active metabolite) leading to higher risk of toxicity	Avoid tramadol use because of potential for toxicity. If opioid use is warranted, consider a non-codeine opioid.
CYP2D6 normal metabolizer	1.25 ≤ × ≤ 2.25	*1/*10 *1/*41, *1/*9 *10/*41 × 3 *1/*1, *1/*2 *2 × 2/*10	Expected O-desmethyltramadol (active metabolite) formation	Use tramadol label recommended age-specific or weight-specific dosing.
CYP2D6 intermediate metabolizer	0 < × < 1.25	*4/*10 *4/*41, *10/*10 *10/*41 *41/*41, *1/*5	Reduced O-desmethyltramadol (active metabolite) formation	Use tramadol label recommended age-specific or weight-specific dosing. If no response and opioid use is warranted, consider non-codeine opioid.
CYP2D6 poor metabolizer	0	*3/*4, *4/*4, *5/*5, *5/*6	Greatly reduced O-desmethyltramadol (active metabolite) formation leading to diminished analgesia	Avoid tramadol use because of possibility of diminished analgesia. If opioid use is warranted, consider a non-codeine opioid.

^a^ is the sum of the values assigned to each allele, which typically ranges from 0 to 3 but may exceed 3 in rare cases. The asterisk (*) symbol is the standard wording to define genotypes.

## 3. Complications Related to Prolonged Treatment

Prolonged drug therapy exposes patients to various side effects. The most common ones are sedation, dizziness, nausea, vomiting, constipation, physical dependence, tolerance, and respiratory depression; less frequent side effects are muscle stiffness, myoclonus, delayed gastric emptying and constipation [180]. Although new molecules have been discovered and new insights into pain mechanisms have been explored over the years, a marked improvement in pain relief has not been achieved yet [181]. Several responsible factors have been identified for therapeutic failure, including the ineffectiveness of pain measurement tools in daily practice, the difficulty of evaluating undertreatment, and the lack of strategies to reduce pain [181].

An issue extensively discussed by the Food and Drug Administration (FDA) and the European Monitoring Centre for Drugs and Drug Addiction (EMCDDA) [182,183] is the growing phenomenon of opioids abuse. The FDA and the Centers for Disease Control and Prevention (CDC) have shown that in the last ten years there has been an increase in overdose deaths and in dependence rates linked to the inappropriate use of these drugs [184,185]. As previously mentioned, opioids provide an analgesic action by binding mainly to µ receptors, which are concentrated in brain regions and control perception of pleasure and well-being, pain perception, and pain-induced emotional responses and reward. Opioids directly activate these brain regions and simultaneously mediate an acquired association between drug intake and the physiological and perceptual effects of the drug, thus producing analgesia and euphoria. Accordingly, repeated opioid intake reinforces these acquired associations and becomes part of the craving for the drug analgesic or pleasurable effects [184]. Therefore, a conditioned need for relief, even from mild pain, can lead to inappropriate use of opioids, which must be discouraged [184]. Furthermore, Butler et al. [184,186] showed that the rewarding effects of opioids are more pronounced when drugs are delivered rapidly into the brain. Indeed, parenteral administration is preferred to achieve non-therapeutic effects and the FDA recommends use of non-injective formulations.

The “ceiling effect” of drugs is the phenomenon whereby an increase in drug administration does not lead to an increase in the pharmacological effect. This phenomenon, typical of many drugs, including weak opioids (codeine and tramadol) and non-opioid analgesics, does not appear to affect strong opioids, except for buprenorphine. This allows a progressive dosage increase in medical practice until sufficient pain control is achieved. However, it is worth underlining that increasing the dosage exposes the patient to the risk of side effects such as sedation, constipation, and respiratory depression [187,188,189].

Tolerance can be defined as a decrease in drug efficacy after repeated or prolonged administration. It can be innate and linked to either pharmacogenetic or acquired characteristics. The latter is determined by reiterated exposure to the drug over time and it depends on a molecular phenomenon involving receptors internalization [190]. A further mechanism responsible for opioid desensitization seems to be the activation of regulatory proteins, such as GPCR kinases, β-arrestins, and adenylate cyclase, capable of “uncoupling” the opioid receptors from the G protein, thus decreasing its analgesic activity [191]. In addition, each opioid has a certain level of “intrinsic efficacy”, a parameter that relates the number of occupied receptors to clinical efficacy; and once analgesia is achieved, the intrinsic efficacy value is inversely proportional to the number of occupied receptors [192,193].

Opioid-induced hyperalgesia is a condition that is clinically revealed by hyperesthesia (markedly increased sensitivity to painful stimuli) and/or allodynia (pain elicited by a normally non-painful stimulus). In hyperalgesia, which can occur in patients undergoing chronic opioid therapy, the perceived pain intensity is heightened, poorly localized and is difficult to define in terms of quality and threshold change tolerability. According to a 2016 study, hyperalgesia occurs in both short-term and long-term treatments, regardless of whether addiction and withdrawal phenomena appear or not [191]. Several mechanisms are associated with opioid-induced hyperalgesia. One of them is the activation of glutamate-associated *N*-methyl-aspartate (NMDA) receptors, which causes sensitization of spinal neurons. In some cases, it has been observed that the receptor antagonists (e.g., ketamine, dextromethorphan, amantadine) block hyperalgesia. Other studies have shown that hyperalgesia is correlated with an increase in excitatory peptide neurotransmitters, such as cholecystokinin (CCK), which, once released in the rostral ventromedial medulla (RVM), activate the spinal pathways that regulate the release of dynorphin. These and other neurotransmitters cause “central sensitization” responsible for a hypersensitivity of the spinal cord to nociceptive inputs from the periphery [191]. If tolerance reflects decreased opioid sensitivity, hyperalgesia represents an increased pain perception. The former is relieved by an increase in opioid dose, while the latter may worsen after an increase in dosage. However, there are many similarities between the mechanisms of tolerance and hyperalgesia; for example, CCK acts on descending pathways that modulate pain, thus contributing to both opioid-induced tolerance and hyperalgesia [190].

Excluding a worsening of the treated pathology, it is unclear if the perception of pain in subjects treated with opioids is the result of reduced treatment efficacy due to tolerance, or to real opioid hyperalgesia. The literature lacks specific studies on humans; however, one study performed on adolescents undergoing surgery for scoliosis and receiving an intraoperative remifentanil infusion demonstrated that these patients required significantly more postoperative morphine than those receiving intermittent morphine boluses, suggesting that the remifentanil infusion was associated with the development of acute clinically relevant opioid tolerance or hyperalgesia [194].

Rotation and switch of therapy are consolidated practices used to overcome the problems related to prolonged treatment with opioid drugs. The first one consists of a shift to another opioid following a pre-set schedule to prevent potential adverse effects and limit dose escalation. The second involves the substitution of one opioid with another due to the appearance of dose-related side effects or inadequate analgesic effect. In both cases, the goal is to achieve equianalgesia, i.e., to maintain the same analgesic power while varying the amount of drug formulation [195]. However, evidence supporting the effectiveness of these methods, which may in some cases be the only option for symptomatic relief, is mostly based on observational studies [188], while there are no studies describing effective opioid rotations [196]. Furthermore, the therapy switch often involves a change in the route of administration, which may have an additional specific effect. To establish its true clinical efficacy more randomized trials are needed. This would also allow us to determine which opioid is more suitable for use in the first or second line and to standardize the conversion ratios when the molecule is switched [196].

Over the past 25 years, one of the main reasons opioids have been increasingly prescribed for the treatment of chronic non-cancer pain is the belief that addiction is a rare consequence of long-term opioid therapy. It is very important to underline that this idea was supported by limited, low-quality data, which are not applicable to current patterns of opioid use. More recent and rigorous studies show that one in three patients undergoing chronic opioid therapy develops a psychological disorder from opioid use and physical dependence occurs in virtually all patients treated with chronic opioid therapy [197,198]. Prescribing opioids to maximize benefits while minimizing harm requires careful assessment of the risks of addiction and its consequences [198,199].

## 4. Therapeutic Drug Monitoring of Opioids, Challenges and Potentials

Therapeutic Drug Monitoring (TDM) is a clinical practice involving the evaluation of drug concentration in a biological matrix (blood, plasma, serum, saliva, and urine) [200,201,202] with the aim of correlating drug concentration with a specific pharmacological activity or toxic effect. Ates and coworkers define TDM as “a dynamic dosing process”, when there is a clear dose-response relationship, and the measurement of drug concentration is used to examine the dosage regimen and thereby evaluate its efficacy or toxicity [200]. Indeed, TDM is a tool that can be routinely used in clinical practice in the field of personalized medicine to establish a tailored drug treatment and help with the therapeutic management of the patient [203].

The TDM approach is not a linear process; the ultimate goal is not solely to determine drug concentrations. It is a complex evolving process of feedback control, which must focus on each individual during therapy [202]. This personalized dynamic nature is related to variations in the drug metabolism in each patient; indeed, the concentrations measurement is a surrogate index of drug exposure in the body [202]. TDM thus requires a series of measurements over time to optimize the drug dosage for the patient [200].

The methodological approaches mainly used for TDM are immunoassays and chromatographic methods [203]. Immunoassays exploit the great affinity between a drug and its target antibody, while chromatographic methods like Ultra-High-Performance Liquid Chromatography (UHPLC), are coupled to special detectors, often mass spectrometry (liquid chromatography coupled to tandem mass LC-MS/MS), but also UV, flame ionization detection (FID) or diode array detection (DAD). These methods are applied on conventional and non-conventional biological matrices, such as blood, plasma, serum, urine, saliva, and interstitial fluid, and are sometimes obtained through unconventional sampling methods such as Dried Blood Spot (DBS), Volumetric Absorptive Microsampling (VAMS), or Salivette^®^, a saliva sampling device [201,204,205]. Hair is also considered as an alternative matrix in which molecules related to the intake of xenobiotic substances may accumulate, allowing us to detect the presence of these molecules even months or years after intake, depending on the length of the hair. Indeed, the keratin matrix has long been used to establish long-term drug abuse and as a test for compliance in clinical toxicology. However, this matrix is not suitable for obtaining quantitative information on drug assumption and the time of intake; therefore, in TDM, it is mainly used to monitor adherence to therapy [206,207].

To be a candidate for TDM, a drug should be used during a period of time sufficient to reach the steady state of drug therapy [200,202], which depends only on the half-life of the drug. Usually, after about five half-lives, more than 95% of a drug will have accumulated and a steady state is reached [202]. The timing between sample collection, the start of the drug dosage, and the last dose administered is crucial when determining the concentration following complete distribution in the tissues and to avoid accumulation in the body [200,202,203]. Drugs with long half-lives can be monitored even before steady state is reached to prevent toxicity at the initial prescribed dosage regimen, in case of known or suspected alterations in metabolism or renal excretion [202].

TDM results may help clinicians to design, optimize, correct, and personalize therapies. Moreover, they provide useful data when a reliable correlation between the clinical effect of a drug and its concentration in the analyzed biological matrix occurs. Furthermore, TDM should be carried out for those active principles, characterized by a narrow therapeutic index, for which easily measurable pharmacological efficacy endpoints are not available and the relationship between dosage regimen and concentration is expected to be poorly predictable because of a marked interindividual variability. However, to understand whether it is appropriate to monitor a drug or its metabolite and to choose the most appropriate biological matrix, it is advisable to consider the pharmacokinetic and pharmacodynamic characteristics of the drug under investigation [200,203]. The selected biological matrix for the TDM should reflect the free (i.e., not bound to proteins) or total drug concentrations; the correlation between drug (or metabolite) levels and concentrations in the biological matrix used should be assessed [203].

As already underlined in this review, the use of opioid analgesics for chronic pain treatment is related to the risk of adverse side effects and also addiction and abuse [208]. Recent studies have attempted to tailor pain relief and related opioid treatment to the patient characteristics, to identify side, toxic, and therapeutic effects [181]. Several methods have been developed for the simultaneous determination of some opioids and their metabolites in plasma and urine by LC-MS/MS [209,210,211].

Although different research groups have evaluated TDM of opioids as a potential clinical strategy for improving pain relief, there is a significant lack of studies in the literature.

Pantano et al. applied their method to determine oxycodone and its main metabolites on a small number of real plasma samples to evaluate the correlation between analgesic effects and plasma concentration [210]. However, although the optimized dosage was determined, only six patients were tested and the study failed to establish an effective relationship between plasma concentration and clinical outcomes [210].

Protti et al. developed an LC-MS/MS-based method to determine the concentrations of oxycodone and its major metabolites in blood and urine matrices, using various sampling approaches and comparing different microsampling procedures. An observational clinical study was performed with eighteen patients with moderate to severe pain who received oral drug-release therapy for at least seven days [211]. In this study, an analytical method for oxycodone quantification was developed and validated. The authors focused on the comparison between different biological matrices and sampling methods to evaluate the relative advantages and disadvantages in the context of TDM and to implement new protocols for anti-doping analysis in athletes. Plasma, dried blood spots (DBS), and dried plasma spots (DPS) have been studied for TDM purposes, while urine, dried urine spots (DUS), and VAMS of urine have been examined with regard to anti-doping [211]. Traditional and miniaturized sampling approaches were comparable with regard to extraction yield. Since all the dried matrices had very low volumes, they involved a considerable advantage in terms of feasibility of the analysis, but this resulted in an overall sensitivity decrease [211].

In the United States, as a consequence of the high and often uncontrolled consumption of opioids, different types of monitoring are currently carried out. Some studies focused on monitoring drug consumption with the aim of identifying misuse and abuse to opioid treatment [208]. Other studies recommend TDM of opioid analgesics to evaluate adherence to maintenance treatment for opioid use disorder [212,213,214]. These suggest that the opioid-assisted treatment (OAT) with buprenorphine could be a front-line medical maintenance intervention for illicit and prescription opioid use disorder. Therefore, in many clinics, opioid medications are dispensed for several days for self-administration and TDM is recommended to monitor adherence with buprenorphine [212,213,214].

Numerous studies for the treatment of chronic pain do not suggest TDM, but a slow titration of opioid drugs [215,216]. Titration is a slow process of searching for the optimal individual dose for analgesia and then adjusting the dosage of a drug, until the desired therapeutic effect is achieved; the main difference between this approach and TDM is that titration is not based on the search for a specific plasmatic concentration of an opioid drug, but rather on the analgesic effect [215]. Unfortunately, the measurement of pain relief is based on pain scales (VRS, NRS or VAS) which are self-assessed by the patient, thus they are affected by the patient’s perception of pain, which might be extremely personal and emotional [1,2,14,15,16,38].

The TDM process, as described above, assumes that there is a definable relationship between drug concentration and pharmacodynamic effects, but for opioids, it is difficult to measure the effect, i.e., pain relief [202]. Therefore, it is difficult to define actual “therapeutic ranges” for these drugs, which are also affected by tolerance and addiction problems. In this light, it would be more feasible to use the TDM approach to define the minimal effective dosage at which patients experience an analgesic effect.

Nevertheless, opioids remain candidates for TDM primarily because these drugs are characterized by high interindividual variability; moreover, in pain therapy, there are often concomitant pathologies and the use of combined pharmacotherapy is frequent, which may increase the risk of potential drug interactions [202].

The TDM for some adjuvant drugs of pain therapy (antiseizures, antipsychotics and antidepressants) is already performed [200,203], while it is not performed for non-narcotic analgesics, since they are not recommended for chronic use.

## 5. Conclusions

In clinical practice, adequate pain management is essential because pain impacts every aspect of the patient’s life. Therefore, effective regimens of pain pharmacological treatment are needed to improve the quality of life. As documented in the studies described in this review, opioids, while widely used, do not lead to optimal analgesia for all patients.

Albeit the European Association for Palliative Care has proclaimed the WHO ladder as essential in the treatment of cancer pain, it has also stated that “there is a shocking lack of evidence to support clinical practice and guidelines at the present time” [216]. Furthermore, to date, the numerous studies and meta-analyses have shown no clear benefit in pain relief for one opioid over the other. Consequently, as an example, there is no consensus on choosing a strong opioid to start with at step 3 of the WHO ladder [217,218,219,220]. The personalization of opioid therapy, the appropriate selection of drug type and the dosage are far from becoming part of the common clinical practice. This is probably a reflection of the relative paucity of data relating to opioid clinical outcomes. The development of opioid treatment must be personalized and adapted to the needs of each patient by integrating different tools. This can be achieved by designing a pathway in which the pain type is accurately assessed from the beginning of drug therapy together with the evaluation of plasmatic drug concentrations by TDM and the evolution of pain relief by scales. All these traits can be coupled to appropriate pharmacogenomic evaluations since, as is already evident for other drugs, the genotyping of enzymes involved in opioid metabolism may be important for the initial evaluation of the personalized therapeutic plan, helping to avoid or significantly reduce potential toxic effects [209,221].

## Data Availability

No new data were created.
